# Metabolic Associated Fatty Liver Disease (MAFLD) and COVID-19 Infection: An Independent Predictor of Poor Disease Outcome?

**DOI:** 10.3390/medicina59081438

**Published:** 2023-08-08

**Authors:** Vladimir Milivojević, Jelena Bogdanović, Ivana Babić, Nevena Todorović, Ivan Ranković

**Affiliations:** 1 Clinic for Gastroenterology and Hepatology University Clinical Centre of Serbia, Dr Koste Todorovica 2, 11000 Belgrade, Serbia; 2 Faculty of Medicine, University of Belgrade, Dr Subotica 8, 11000 Belgrade, Serbia; 3 Clinic for Endocrinology, Diabetes and Metabolic Diseases University Clinical Centre of Serbia, Dr Subotica 13, 11000 Belgrade, Serbia; 4 Clinic for Infectious and Tropical Diseases University Clinical Centre of Serbia, Bulevar Oslobođenja 16, 11000 Belgrade, Serbia; 5 Department of Gastroenterology, Royal Cornwall Hospitals NHS Trust, Truro TR1 3LJ, UK; doctorranke@gmail.com

**Keywords:** COVID-19, MAFLD, disease outcome, severity

## Abstract

*Background and Objectives*: Early reports on COVID-19 infection suggested that the SARS-CoV-2 virus solely attacks respiratory tract cells. As the pandemic spread, it became clear that the infection is multiorganic. Metabolic associated fatty liver disease (MAFLD) is a chronic liver disease strongly associated with insulin resistance and diabetes. The aim of this study was to assess a possible interplay between MAFLD and COVID-19 infection and its implication in COVID-19 outcome. *Materials and Methods*: A retrospective observational study, including 130 COVID-19 positive patients was conducted. MAFLD diagnosis was made based on the International Consensus criteria. Patients were divided into two groups, group A (MAFLD) and group B (nonMAFLD). Anthropometric and laboratory analysis were obtained. COVID-19 severity was assessed using the NEWS2 score. Disease outcome was threefold and regarded as discharged, patients who required mechanical ventilation (MV), and deceased patients. *Results*: MAFLD prevalence was 42%, 67% of patients were discharged, and 19% needed MV. Mortality rate was 14%. MAFLD patients were significantly younger (*p* < 0.001), and had higher body mass index (*p* < 0.05), respiratory rate (*p* < 0.05) and systolic blood pressure (*p* < 0.05) than nonMAFLD patients. Regarding metabolic syndrome and inflammatory markers: group A had significantly higher glycemia at admission (*p* = 0.008), lower HDL-c (*p* < 0.01), higher triglycerides (*p* < 0.01), CRP (*p* < 0.001), IL-6 (*p* < 0.05) and ferritin (*p* < 0.05) than group B. MAFLD was associated with more prevalent type 2 diabetes (*p* = 0.035) and hypertension (*p* < 0.05). MAFLD patients had a more severe disease course (NEWS2 score, 6.5 ± 0.5 vs. 3 ± 1.0, *p* < 0.05). MAFLD presence was associated with lower patient discharge (*p* < 0.01) and increased need for MV (*p* = 0.024). Multiple regression analysis showed that BMI (*p* = 0.045), IL-6 (*p* = 0.03), and MAFLD (*p* < 0.05) are significant independent risk factors for a poor COVID-19 outcome. *Conclusions*: The prevalence of MAFLD is relatively high. MAFLD patients had a more severe COVID-19 clinical course and worse disease outcome. Our results imply that early patient stratification and risk assessment are mandatory in order to avoid poor outcomes.

## 1. Introduction

Coronavirus disease 19 (COVID-19) is a disease that originated in China in December of 2019 and has, by now, reached a pandemic level [1]. It is caused by a pathogen, a novel coronavirus called the severe acute respiratory syndrome coronavirus (SARS-CoV-2). Similar infections from the same family of viruses have been noted before (SARS and MERS in 2003 and 2012, respectively), both reaching epidemic levels [2]. SARS-CoV-2 infection begins with the viral spike protein’s attachment to an adequate host cell receptor. It has been shown that angiotensin-converting enzyme 2 (ACE2) is the prime surface protein that enables virus cell entrance [3]. However, the SARS-CoV-2 spike protein harbors a single mutation that significantly increases its affinity towards the ACE2 receptor, thus suggesting that this novel virus has evolved with an increased ability to spread among humans [4].

Early reports focused solely on respiratory tract involvement, but as the pandemic spread and further investigation was conducted, it became clear that COVID-19 affects other organs as well [5]. Accumulating evidence suggests that while the virus primarily affects the lungs, it can also affect other organs, causing systemic consequences, coagulopathy and multiple organ injuries. The etiology of the systemic effects of COVID-19 is multifold.

It has been suggested that there are several other receptors and coreceptors that facilitate virus entry, thus enabling one of the possible systemic effect mechanisms of SARS-CoV-2. Liver tropism of SARS-CoV-2 has been postulated before, considering ACE2 receptors have been found in the liver during biopsy [6,7]. However, mechanisms of liver injury in COVID-19 infection are multifold, including direct cytopathic effect, drug-induced injury, hypoxic injury, and cytokine-storm-enhanced inflammation in patients with chronic liver disease (CLD) [7]. The relationship between CLD and COVID-19 seems to be bidirectional, with COVID-19 worsening CLD through its systemic complications while at the same time, compromised liver function in patients with CLD may lead to adverse events in patients with concomitant COVID-19 infection [7].

Fatty liver disease (SLD) as a CLD was first described in 1886 and later further divided into alcoholic liver disease (ALD) and non-alcoholic fatty liver disease (NAFLD) [8]. By global consensus, a novel term of metabolic associated fatty liver disease (MAFLD) was proposed as a way of lifting the stigma off of patients with ALD, considering MAFLD is an inclusive diagnosis made based on the presence of several metabolic syndrome parameters, including obesity and/or diabetes [9]. Some scientists regard MAFLD as a hepatic complication of diabetes, considering the role of insulin resistance, low-grade inflammation, and oxidative stress in its pathogenesis [10]. MAFLD covers a broad spectrum of different disease phenotypes, from simple liver steatosis to fibrosis and ultimately liver failure [8].

Considering that previous studies have identified diabetes and obesity as risk factors for COVID-19 infection and having in mind that both diabetes and obesity, by definition, can accompany MAFLD, it places these patients in a vulnerable, COVID-19-susceptible group, potentially prone to adverse clinical outcomes. However, it is still controversial whether MAFLD is a causal factor in promoting the progression of COVID-19 infection or the said infection exacerbates the existing chronic disease, which in turn leads to poor disease outcome.

In that sense, the aim of our study was to assess a possible interplay between MAFLD and COVID-19 infection and its implication in COVID-19 outcomes.

## 2. Materials and Methods

### 2.1. Study Design

A retrospective observational study was conducted. A total of 130 patients treated at a tertiary health care center from June 2021 until November 2021 were included. 

A COVID-19 infection diagnosis was made following a positive RT-PCR nasopharyngeal swab test. Patients were divided into two groups according to the presence of MAFLD. MAFLD diagnosis was made based on the international consensus criteria [9]. Hepatic steatosis was detected by imaging techniques (ultrasound of the abdomen) and blood biomarkers (fatty liver index, FLI). Patients were measured for waist circumeference and body mass index (BMI, kg/m^2^), and classified as normal weight (<25 kg/m^2^), overweight (25–29.9 kg/m^2^), or obese (>30 kg/m^2^). FLI was calculated by using the BMI, waist circumference, serum triglycerides, and gamma-glutamyl transferase (g-GT) levels. Type 2 diabetes (T2D) was diagnosed based on the patient’s medical history or defined as: 1. fasting plasma glucose ≥ 7 mmol/L (126 mg/dL), 2. random plasma glucose ≥ 11.1 mmol/L (200 mg/dL), 3. HbA1c ≥ 7%, according to the American Diabetes Association (ADA) criteria [11]. Atherosclerotic cardiovascular disease (ASCVD) was regarded as the presence of coronary heart disease (CHD) such as myocardial infarction, angina or coronary artery disease, cerebrovascular disease, or peripheral artery disease [12]. Chronic kidney disease (CKD) was defined as decreased glomerular filtration (GFR) of less than 60 mL/min/1.73m^2^ for at least three months [13]. Upon admission, vital parameters, including heart rate (HR), respiratory rate (RR), oxygen saturation (sO2), systolic (SBP) and diastolic blood pressure (DBP), body temperature (t), and state of consciousness, were assessed.

COVID-19 severity was determined using the National Early Warning Score 2 (NEWS2). The initial score, NEWS, was developed in 2012 by the Royal College of Physicians (RCP) in order to detect all-cause deterioration and predict disease outcome [14]. It comprises RR, oxygen saturation, SBP, body temperature, and state of consciousness. An upgraded version, NEWS2, differs from the previous in terms of the addition of new-onset confusion and a new oxygen saturation scale for hypercapnic respiratory failure (scale 2) [15]. Each category is scored 0–3 and combined to give an overall score with two and three additional points for the use of supplemental oxygen and altered state of consciousness (alert, verbal, pain, unresponsive (respectively). A total score ranges from 0 to 20, with patients being divided into three categories based on the clinical risk assessed: low (aggregate score 0–4), medium (aggregate score 5–6), and high (aggregate score 7 or more). If the patient scores 3 in any individual category, they are immediately classified as high-risk. It has been demonstrated that a higher NEWS2 score is a good predictor of short-term mortality in COVID-19 patients [16].

Disease outcome was regarded as: 1. discharged patients (survivors, not treated in the Intensive Care Unit (ICU)), 2. patients needing mechanical ventilation (MV), thus being transferred to the ICU, and 3. deceased patients (non-survivors).

### 2.2. Inclusion and Exclusion Criteria

Inclusion criteria consisted of COVID-19-positive consecutive patients aged > 18 years old. Exclusion criteria were pregnancy, previous viral hepatitis, cirrhosis, liver tumors (benign or malignant), or ALD.

### 2.3. Measurements

Data on comorbidities and vital, anthropometric, and biochemical parameters were obtained.

Assessed vital parameters were: RR (n/min), HR (n/min), SBP (mmHg), DBP (mmHg), body temperature (C), oxygen saturation (%), and state of consciousness (AVPU).

BMI was measured according to the equation weight (kg)/height (m^2^). Biochemical data consisted of metabolic syndrome parameters taken after an 8 h fasting (glycemia (mmol/L and mg/dL), triglyceride levels (mmol/L and mg/dL) and HDL-cholesterol (mmol/L and mg/dL)) and synthetic liver function markers (AST (U/L), ALT (U/L), g-GT (U/L), ALP (U/L), albumins (g/L), and platelets (g/L)). The De Ritis coefficient was measured as the AST/ALT ratio and inflammatory markers (C-reactive protein (CRP) (mg/L), fibrinogen (g/L), ferritin (ug/L), and interleukin-6 (IL-6) (pg/mL)).

### 2.4. Ethical Compliance

Considering this is a retrospective study, all discharged participants were informed of the details of the study and signed an informed consent form for participation (regarding their medical data) in accordance with the Declaration of Helsinki. Regarding the medical dataset of the deceased patients, their representative family members were informed of the study details and signed an informed consent form. All procedures were conducted in accordance with standard clinical settings. The study was approved by the Ethics Committee of the University Clinical Centre of Serbia.

### 2.5. Statistical Analysis

Descriptive statistical analyses were performed. The normality test was performed for numerical variables and based on the test results, numerical variables were presented as means and standard deviation (SD). Categorial variables were presented as percentages (%). Comparisons of anthropometric and clinical variables between two groups were conducted using the Student *t*-test (for continuous variables) and the Chi square test (for categorical variables). The correlation was assessed using the Pearson correlation coefficient (r) for continuous variables. Univariate and multivariate logistic regression analyses were used to determine factors for disease outcome. The odds ratio (OR) and relative risk (RR) were measured with death and need for mechanical ventilation being used as a composite poor disease outcome. Results were expressed as OR and RR (respectively), with a corresponding 95% confidence interval (CI). The level of significance was set at 0.05. All statistical analyses were performed using SPSS 21.0. 

## 3. Results

**Figure 1:**
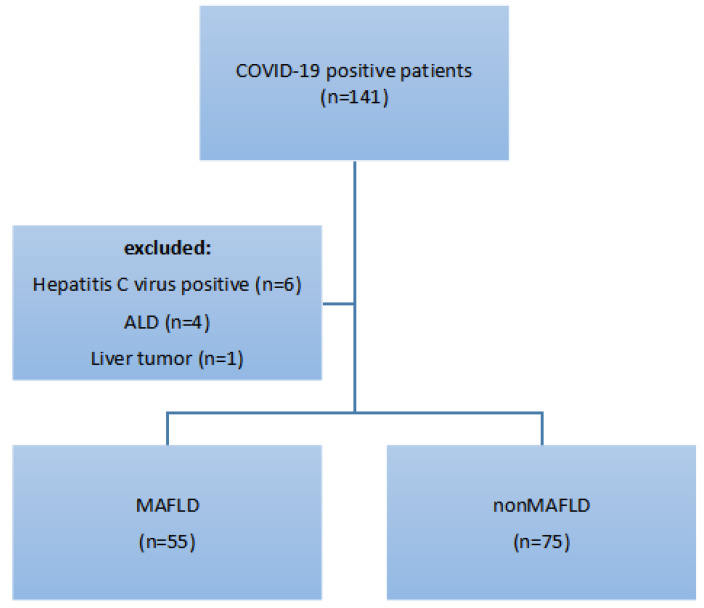
Inclusion process.

Among 130 COVID-19-positive patients, based on the international consensus criteria, 42% (n = 55) were placed in the MAFLD group (group A), while 58% (n = 75) of patients were placed in the nonMAFLD group (group B). The selection process is presented in Figure 1.

Concerning anthropometric parameters, age, gender, and BMI were evaluated. There was no statistically significant difference between groups concerning gender (male/female, 29/26 vs. 40/35, *p* = 0.94). MAFLD patients were significantly younger (53.5 ± 4.5 vs. 62.3 ± 4.2, *p* < 0.001) and had a higher BMI (28.5 ± 2.8 vs. 24.8 ± 3.1, *p* < 0.001), as seen in the Table 1.

Having assessed vital parameters, we noticed that MAFLD patients had a higher SBP (142.5 ± 10.1 vs. 130.4 ± 7.9, *p* < 0.001) while there was no difference considering DBP, RR, or HR (Table 1).

**Figure 2:**
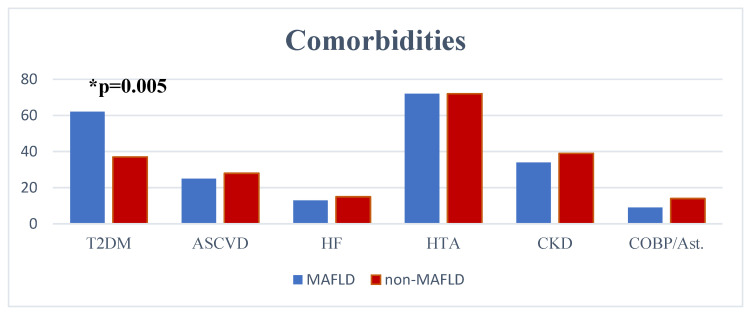
Comorbidites prevalence comparison.

In order to evaluate the comorbidities-related differences between groups, we evaluated the prevalence and potential differences regarding T2D, ASCVD, heart failure (HF), arterial hypertension, CKD, and chronic obstructive pulmonary disease or asthma (COPD). It was noted that T2D was significantly more prevalent in the MAFLD group (63% vs. 37%, *p* = 0.005). Regarding other comorbidities, no significant difference between groups was observed (Figure 2).

Evaluation of liver function parameters showed significant in-between-group differences regarding aspartate aminotransferase (AST), alanine aminotransferase (ALT), gamma glutamyl transferase (g-GT), alkaline phosphatase (ALP), platelets, and albumin levels. Group A had significantly higher levels of AST (52.3 ± 11.2 vs. 45.5 ± 8.9, *p* = 0.002), ALT (64.4 ± 12.5 vs. 47.5 ± 10.2, *p* = 0.001), and ALP (108.3 ± 10.3 vs. 101.4 ± 13.3, *p* = 0.001) as presented in the Table 2. Simultaneously, no significant difference was noted regarding g-GT. On the other hand, the MAFLD group had significantly lower platelets (145 ± 14.2 vs. 179.5 ± 11.6, *p* < 0.001) and albumin levels (31 ± 4.5 vs. 38.5 ± 5.1, *p* < 0.001). A between-group difference was also observed for the AST to ALT (AST/ALT) ratio (De Ritis ratio), with a lower level in the MAFLD group (0.84 ± 0.04) as opposed to the nonMAFLD group (0.91 ± 0.03) (*p* < 0.001) (Table 2).

Having assessed inflammatory markers, we observed a significant difference regarding C-reactive protein (CRP), interleukin-6 (IL-6), and ferritin, with the MAFLD group having higher levels of the aforementioned markers compared with nonMAFLD patients (CRP: 29.5 ± 11.0 vs. 21.2 ± 7.9, *p* < 0.001, IL-6: 56.5 ± 5.1 vs. 54.2 ± 4.1, *p* < 0.05, ferritin: 331.2 ± 31.0 vs. 256.2 ± 28.5, *p* < 0.05). There was no significant in-between-group difference regarding fibrinogen levels (4.6 ± 1.2 vs. 4.5 ± 1.1, *p* = 0.62) as shown in Table 3.

Concerning metabolic syndrome parameters, MAFLD patients had higher triglycerides (Tg) (59.4 ± 16.2) and fasting glycemia levels (151.2 ± 21.6) as compared with the nonMAFLD group: Tg 50.4 ± 18.0 (*p* < 0.001), glycemia 127.8 ± 46.8 (*p* = 0.008). At the same time, HDL-cholesterol (HDL-c) levels were lower in group A than in group B (12.6 ± 1.8 vs. 18.0 ± 7.2, *p* < 0.01) (Table 4).

Analysis of disease severity was made using the validated NEWS2 score, with higher values indicating higher risk of COVID-19 severity and expressed as mean ± SD. 

**Figure 3:**
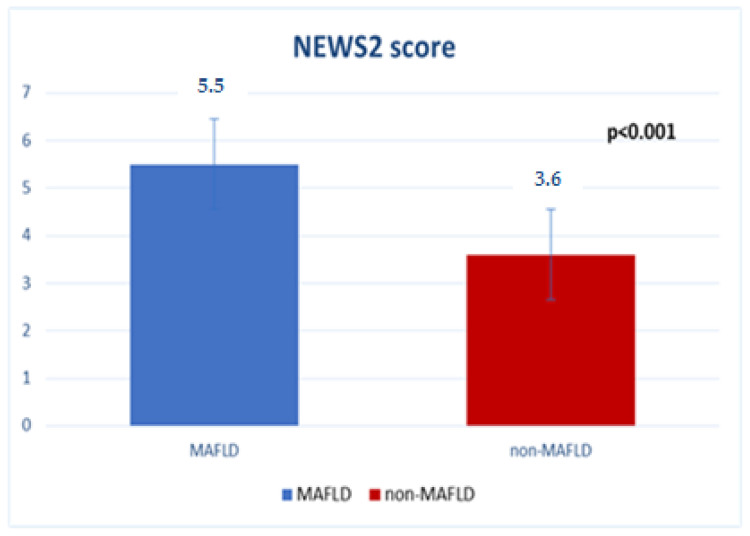
Disease severity: comparison.

Results showed that MAFLD patients had a higher NEWS2 score (5.5 ± 1.1) than nonMAFLD patients (3.6 ± 0.9) (*p* < 0.001), as presented in Figure 3.

**Figure 4:**
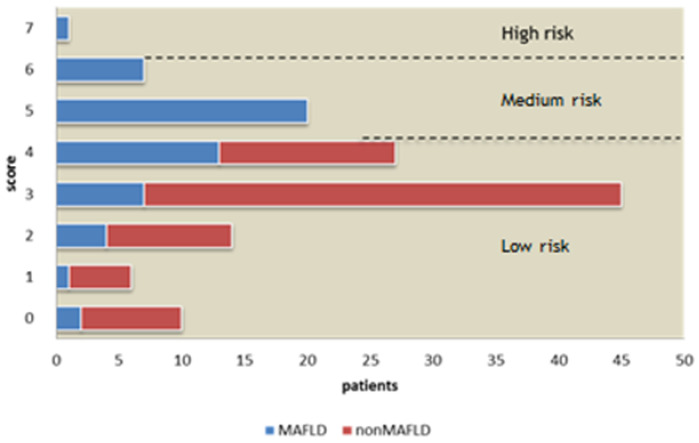
Patient stratification in risk-groups according to NEWS2 score: comparison.

Further subanalysis of disease severity by dividing patients into low, medium, and high risk groups revealed a gradual increase in the number of MAFLD patients in the medium and high risk groups as compared to the low risk patient group. Results presented in Figure 4 indicate only MAFLD patients were stratified into medium and high risk groups.

**Figure 5:**
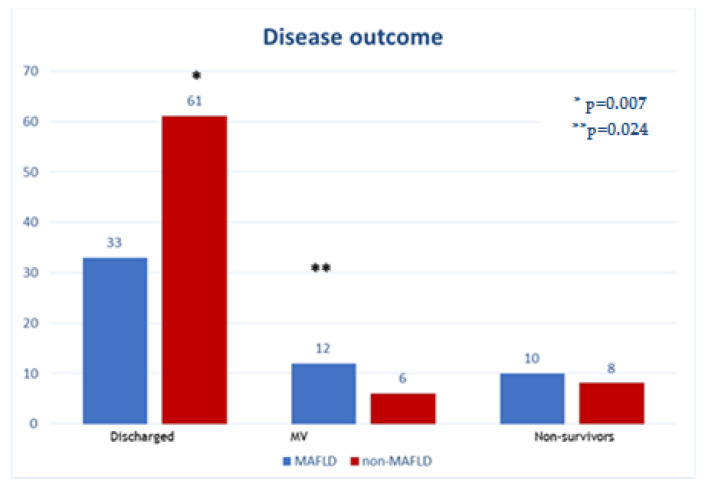
Individual disease outcome parameters: comparison.

Disease outcome was threefold and was regarded as discharged patients, patients needing MV, and deceased patients. Group A had a lower number of discharged patients than Group B (60% vs. 81%, *p* = 0.007). Further, MAFLD patients had a higher need for MV than nonMAFLD patients (22% vs. 8%, *p* = 0.024), while no significant difference was noted concerning the death rate (18% vs. 11%, *p* = 0.24) between groups (Figure 5). In further analysis, a composite outcome of need for MV and death was used as a marker for poor disease outcome.

**Figure 6:**
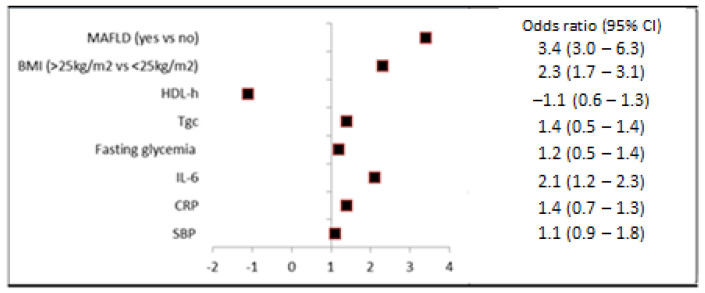
Regression analysis: odds ratio for predictors of COVID-19 outcome.

Using univariate logistic regression analysis, MAFLD presence, BMI, HDL-c, Tg, SBP, and IL-6 were found to be significantly associated with poor disease outcome. In the final multivariate regression analysis, three predictors of poor COVID-19 outcome, namely MAFLD (odds ratio (OR) 3.4, 95% confidence interval (CI) 3.0–6.3; *p* < 0.001), BMI (OR 2.3, 95% CI 1.7–3.1; *p* = 0.045), and IL-6 (OR 2.1, 95% CI (1.2–2.3); *p* = 0.03), remained significant (Figure 6, Table 5). In order to avoid overestimating the effect of MAFLD presence on poor COVID-19 outcomes, a risk ratio was further calculated. A relative risk of MAFLD patients having a poor disease outcome was 2.1 (95% CI 1.2–3.8, *p* < 0.001).

## 4. Discussion

In the present study, we provide vital insight into the prevalence of MAFLD and its possible association with COVID-19 severity and outcome.

Metabolic syndrome represents a cluster of diseases such as type 2 diabetes, obesity, hypertension, and dyslipidemia [17]. All of these conditions have been proven to pose a risk for poor outcome of COVID-19 infection [17,18]. MAFLD is often regarded as a hepatic complication of diabetes and, in that sense, may contribute to the development of a more severe form of COVID-19 [19].

In our retrospective observational study, MAFLD was diagnosed in 42% of COVID-19-positive patients. The prevalence of MAFLD has been steadily growing in the past two decades, from 25.5% before 2005 to 38.9% in 2020, with the highest prevalence reported in the Middle East (31.79%) and South America (30.45%) and the lowest in Africa (13.48%) [20].

It is thought that the increasing burden of other metabolic diseases such as type 2 diabetes (T2D), dyslipidemia, and obesity is the main driving force behind the increase in MAFLD prevalence. Notably, in the last two years, concurrent with the global COVID-19 pandemic, a much greater increase (2.16 annual rate of change) in the prevalence of MAFLD has been reported [21]. It is estimated that by 2040, the prevalence of MAFLD will be 55.7%, which is a three-fold increase since 1990 and a 43.2% increase from the 2020 prevalence of 38.9% [21]. As of now, MAFLD is the most common chronic liver disease (CLD) and a leading cause of liver-related morbidity and mortality [22].

The diagnosis of MAFLD in the setting of COVID-19 infection is challenging, considering numerous studies have shown an increase in liver enzyme levels during COVID-19 infection [6,7,23]. It is still not fully understood what the driving mechanism behind this process is. Noticeably, in our study MAFLD patients had significantly elevated hepatocellular dysfunction markers. This is interesting considering our findings dominantly point to liver injury on a hepatocellular level. However, previous studies on extrapulmonary manifestations of COVID-19 and conducted liver biopsies proved ACE2 receptors were dominantly present on cholangiocytes [7]. Nevertheless, this could be explained by the multifold nature of COVID-19-mediated liver injury [23]. Additionally, this can be attributed to the condition itself, considering elevated liver enzymes are part of the MAFLD diagnostic criteria. Patients with preexisting liver disease (LD) are more susceptible to disease deterioration during an infection, with COVID-19 being one of the most challenging ones [24]. However, this phenomenon was also noted in patients without previous liver dysfunction [25]. It has been postulated that an uncontrolled immune response with a high release of cytokines causing hyperinflammation and consequently multi-organ damage may be one of the possible causators [23]. On the other hand, metabolic diseases, such as MAFLD, T2D, and obesity, cause low-grade inflammation through certain proinflammatory cytokines such as TNFalpha and IL-6, which can act as a steppingstone in a vicious cycle of hyperinflammation and cytokine storm, resulting in target organ damage [7]. In highly specific circumstances, such as COVID-19 infection, in order to exclude confounding factors in diagnosing MAFLD, imaging techniques such as ultrasound and CT scan are of great help and should be used as diagnostic tools alongside other criteria as proposed by the International Consensus Report Guidelines [9].

The presence of MAFLD was evaluated upon patient admission and regarded as a newly diagnosed condition for the majority of our patients, highlighting the fact that most of our patients were unaware of LD presence even when harboring multiple risk factors. Studies have shown a lack of awareness regarding the prevalence and diagnosis of MAFLD, which suggests the need for large-scale implementations of educational programs [26].

In our study, MAFLD patients were significantly younger and expectedly had a higher BMI. However, mean BMI did not reach the obesity grade level. Nevertheless, it has been reported that not only obese patients but also overweight patients are at a higher risk of developing a severe form of COVID-19 and needing respiratory support as well as invasive ventilation [27]. The patophysiological mechanisms underlying the association between BMI and COVID-19 severity are multifold and probably a result of the bidirectional relationship between virus and host impairment [28]. The immunomodulatory effect of chronic low-grade inflammation present in overweight people simultaneously with suboptimal T-cell and B-cell responses and possible dysfunctional respiratory capacity in overweight people suggests the possibility of invasive ventilation susceptibility [29]. Studies have shown that overweight and obesity are independent risk factors for a critical clinical course of COVID-19 infection even in the young population, which is in line with our findings, considering our MAFLD patients were significantly younger than the nonMAFLD patient group [30].

Our patients had a higher respiratory rate (RR) and a higher systolic blood pressure (SBP). Both of these parameters have been shown to indicate a poor prognosis in sepsis-related critical conditions [31]. It has been shown that RR is an indicator of lower respiratory tract infection and is deemed an important tool for assessing disease severity in clinical settings [32]. Additionally, it has been shown that not only arterial hypertension as a chronic disease but also acutely elevated SBP values were associated with poor disease outcome [33]. High SBP was identified as a covariate in both mortality and survival prediction models and was present in deceased COVID-19 patients as compared to discharged individuals [33]. Even though there was no statistical difference noted in the presence of hypertension in our patient groups, it is worth noting that elevated SBP could be a marker of pre-existing hypertension-mediated subclinical organ damage (HMOD, i.e., vascular stiffness), thus representing an important comorbidity factor [34]. Higher SBP could also be the consequence of reduced enzymatic activity of ACE2 caused by the binding of a higher SARS-CoV-2 load [35].

Secondary to hypertension, type 2 diabetes (T2D) was proven to be the most common comorbidity in patients with COVID-19 infection as well as the most common concomitant disease in patients with MAFLD [18,36]. In keeping with that, in our study, T2D was significantly more prevalent in MAFLD than in the nonMAFLD group. The impact of T2D on COVID-19 severity and outcome still remains a controversial topic. Early reports have proposed diabetes as a major risk factor for COVID-19 development. A Chinese meta-analysis showed a 9.8% prevalence of diabetes among COVID-19 hospitalized patients, which is the equivalence of overall diabetes prevalence in China [37]. In a similarly structured large study conducted in the UK, 32% of participants had T2D [38]. However, it was later established that diabetes per se does not contribute to COVID-19 susceptibility, but poorly controlled diabetes and acute hyperglycemia are risk factors for hospital admission, disease severity, and its poor outcome [39]. Recent meta-analyses showed that diabetes increases the risk of severity by three times, and two-and-a-half times the risk of COVID-19-associated death [40]. Studies have shown that even short-term hyperglycemia can cause an impaired immune system response and compromise both innate and adaptive mechanisms of action [41]. Moreover, diabetes causes decreased expression of ACE2, which in turn has antioxidative capacity and lowers inflammation, thus making COVID-19 diabetics more susceptible to hyper-inflammation and cytokine storm [42].

It is established that insulin resistance can be regarded as a cornerstone for fatty liver development [36]. Perturbations in insulin action lead to increased liver lipid accumulation, a process called lipotoxicity. While assessing lipid status of our patients, lipid abnormalities regarding triglycerides (Tg) and HDL-cholesterol (HDL-c) were noted. MAFLD patients had a significantly higher level of Tg and a significantly lower level of HDL-c as opposed to the nonMAFLD group. Recently, studies have emerged proposing that atherogenic dyslipidemia be used as a predictor of the critical COVID-19 course [43]. Apart from being closely linked to insulin resistance and diabetes, whose pathophysiological mechanisms of damage in COVID-19 were previously discussed, atherogenic dyslipidemia itself, regardless of diabetes status, may play a role in poor clinical outcome in COVID-19 hospitalized patients. In a retrospective Italian study, Bellia et al. found atherogenic dyslipidemia to be significantly more prevalent in critically ill patients and was associated with mortality in both the diabetes cohort and overall population of COVID-19 hospitalized patients [43]. This association was more potentiated if paired with visceral and/or overall obesity, as marked by the BMI. This is in accordance with our results, considering MAFLD patients were predominantly overweight and had a significantly higher BMI than nonMAFLD group. However, when adjusted for sex, age, and other confounding factors, Tg and HDL-c did not show significant contributions regarding disease course and outcome.

Regarding pro-inflammatory markers, MAFLD patients had significantly higher levels of CRP and ferritin. CRP is an acute-phase protein synthesized in the liver and is expectedly elevated in inflammatory states such as infection or tissue injury [44]. Considering MAFLD is closely related to greater derangements in the metabolic profile and linked to low-grade inflammation, it has been postulated that CRP can potentially be used as a valuable tool in predicting its progression. CRP is a proven independent risk factor for cardiovascular and all-cause mortality in a number of chronic diseases, LD included [45]. However, its role in predicting liver-related morbidity and mortality remains controversial [46]. Even though MAFLD patients had higher CRP levels than the nonMAFLD group, in further analysis, CRP failed to reach statistical significance regarding its independent contribution to COVID-19 outcomes. Furthermore, ferritin is another acute phase reactant that has emerged as a potential marker for MAFLD disease activity considering insulin resistance, inflammation, and steatosis (all found in MAFLD) lead to an altered iron metabolism, thus causing elevated ferritin levels [47]. Numerous studies have confirmed increased ferritin in patients with MAFLD/NAFLD [48]. Kowdley et al. showed an independent association between ferritin level and increased risk for liver fibrosis in patients with NAFLD [48]. On the contrary, two large retrospective studies concluded that even though ferritin was associated with liver steatosis, it was a poor predictor of fibrosis stage and disease progression [49]. This was in keeping with our results. Both MAFLD and nonMALD patients had ferritin levels above the reference range, with the MAFLD group having significantly higher levels. However, that could be contributed to the over-inflammation response caused by the SARS-CoV-2 virus targeting already inflamed liver tissue. Further analysis excluded ferritin as an independent predictor of COVID-19 outcome.

It is widely accepted that cytokines play a critical role in the pathogenesis of MAFLD by activating various signaling pathways that interfere with insulin signaling [50]. Pro-inflammatory cytokines secreted by adipose tissue involved in this process include, among others, interleukin-6 (IL-6). It has been postulated that IL-6 plays a critical role in virus-induced cytokine storm, hence, a recombinant humanized monoclonal antibody IL-6 receptor inhibitor, tocilizumab, has recently emerged as an alternative treatment for COVID-19 [51]. On the other hand, considering MAFLD is closely related to immunologically activated adipose tissue, studies have shown IL-6 levels to be elevated in patients with this condition [52]. Our study confirmed what was expected: all COVID-19 patients had increased IL-6, with MAFLD patients expressing significantly higher levels. This could be attributed to IL-6 being a marker of inflamed liver and/or adipose tissue. Even though the MAFLD group had a higher BMI, robustly indicating a higher source of adipose tissue as a possible explanation for the difference in IL-6, studies have shown that regardless of body fat percentage, MAFLD is associated with higher IL-6 [53]. It has been noted that in the setting of COVID-19 infection, IL-6 has additive potential for indicating disease severity [54]. In that sense, we noted that IL-6 was an independent predictor of disease outcome, with higher levels of the cytokine corresponding to higher mortality and mechanical ventilation as markers for disease outcome.

Following the first reports of certain patient groups suffering rapid health deterioration and needing invasive respiratory support, it became clear that patient stratification would be mandatory. Efforts have been made in order to produce the best tool possible for early patient triage.

The National Early Warning Score (NEWS) had been recommended by the Royal College of Physicians in 2012 as a standardized track and trigger early warning system to grade acute illness severity, detect acute clinical deterioration, and help guide clinical decision-making [14]. An updated version of NEWS, NEWS2, was developed in 2017 and, by 2020, was widely used across the UK for early disease stratification in acutely ill patients with undifferentiated illness or sepsis. In the setting of COVID-19, NEWS2 showed superiority to other similarly constructed scores, with high sensitivity and specificity in early risk assessment [16].

MAFLD patients showed an overall higher score rating upon admission as opposed to the nonMAFLD group, with higher ratings in almost every NEWS2 parameter. Noticeably, the prevalence of nonMAFLD patients was significantly higher in the low clinical risk group. However, there were zero nonMAFLD patients stratified into medium and high clinical risk.

Regarding clinical outcome, MAFLD patients more significantly showed the need for mechanical ventilation, while nonMAFLD patients were more frequently discharged. There was no significant difference regarding death as a clinical outcome. When assessing a composite outcome consisting of MV need and death, again, the nonMAFLD group showed favorable results.

Assessing individual risk factors, MAFLD presence, higher BMI, and elevated IL-6 showed statistically significant unfavorable effects of the COVID-19 outcome.

Based on our results, poor clinical course and worse disease outcome of MAFLD patients could be attributed to various factors that, unfortunately, in this vulnerable patient group seem to act simultaneously. Higher cardiometabolic risk (reflected by dyslipidemia, dysglycemia, and higher BMI), low grade inflammation (further potentiated by the COVID-19 infection) and the burden of having more than one chronic-metabolic-dysfunction associated disease, contribute to further compromisation of patients’ immune defense mechanisms, ultimately leading to worse COVID-19 outcome.

Our study has certain limitations. It is an observational retrospective study; hence, a certain level of bias is hard to avoid. Direct patient comparison was made at the beginning of their hospital stay and hence may not have reflected the interrelationship between MAFLD and COVID-19 in the best way. Additionally, a moderate number of MAFLD patients were included. However, to the best of our knowledge, this is the first study reporting Serbian experience regarding MAFLD prevalence and association with COVID-19 course and outcome.

## 5. Conclusions

MAFLD prevalence has been on the rise in the last two decades following the increase in global prevalence of other closely related metabolic disorders. Immunomodulatory effects, hyper-inflammation, and chronic oxidative stress have made this already vulnerable population more susceptible to respiratory derangement. COVID-19-positive patients with MAFLD are at a higher risk of developing a more severe form of the disease as well as a worse disease outcome. Early patient stratification with the assessment of independent risk factors is mandatory in order to make specific recommendations, and provide guidance on therapy, all in order to ultimately achieve better treatment results.

## Figures and Tables

**Table 1:** Anthropometric and vital parameters comparison at admission.

Anthropometric and Vital Parameters	MAFLD	nonMAFLD	*p* Value
Gender (male vs. female)	29/26	40/35	0.94
Age (years)	53.5 ± 4.5	62.3 ± 4.2	<0.001
Body mass index (BMI, kg/m^2^)	28.5 ± 2.8	24.8 ± 3.1	<0.001
Respiratory rate (RR, n/min)	19.5 ± 3.1	16.2 ± 4.1	<0.001
Heart rate (HR, n/min)	89.1 ± 14.2	82.1 ± 11.9	0.6
Systolic blood pressure * (>140 mmHg, yes)	14	21	<0.05
Diastolic blood pressure * (<90 mmHg, yes)	7	11	0.43
Body temperature	37.4 ± 1.1	37.1 ± 0.9	0.09
Oxygen saturation * (supplemental oxygen, yes)	14	12	0.52

* number of patients.

**Table 2:** Liver function markers: comparison.

Liver Function Parameters	MAFLD	nonMAFLD	*p* Value
AST (U/L)	52.3 ± 11.2	45.5 ± 8.9	0.002
ALT (U/L)	64.4 ± 12.5	47.5 ± 10.2	<0.001
ALP (U/L)	108.3 ± 10.3	101.4 ± 13.3	0.0017
g-GT (U/L)	40.4 ± 11.0	41.5 ± 9.9	0.55
De Ritis coefficient	0.84 ± 0.04	0.91 ± 0.03	<0.001
Albumins (g/L)	31 ± 4.5	38.5 ± 5.1	<0.001
Platelets (g/L)	145 ± 14.2	179.5 ± 11.6	<0.001

Notes: upper normal limits: AST 37 U/L, ALT 41 U/L, g-GT 38 U/L, ALP 120 U/L, albumins 53 g/L, platelets 424 × 10^9^/L.

**Table 3:** Inflammatory markers: comparison.

Inflammatory Markers	MAFLD	nonMAFLD	*p* Value
CRP (mg/L)	29.5 ± 11.0	21.2 ± 7.9	<0.001
IL-6 (pg/mL)	56.5 ± 5.1	54.2 ± 4.1	<0.05
Ferritin (μg/L)	331.2 ± 31.0	256.2 ± 28.5	<0.05
Fibrinogen (g/L)	4.6 ± 1.2	4.5 ± 1.1	0.62

Notes: upper normal limits: CRP 5.0 g/L, IL-6 7.0 pg/mL, ferritin 150.0 ug/L, fibrinogen 4.0 g/L.

**Table 4:** Metabolic syndrome parameters: comparison.

Metabolic Syndrome Parameters	MAFLD	nonMAFLD	*p* Value
HDL-cholesterol (mg/dL)	12.6 ± 1.8	18.0 ± 7.2	<0.01
Triglycerides (mg/dL)	59.4 ± 16.2	50.4 ± 18.0	<0.001
Fasting glycemia (mg/dL)	151.2 ± 21.6	127.8 ± 46.8	0.008
SBP (mmHg)	142.5 ± 10.1	130.4 ± 7.9	<0.001
DBP (mmHg)	82.2 ± 5.6	83.0 ± 5.8	0.43

**Table 5:** Multivariate regression analysis: predictors of COVID-19 outcome.

Variables	B	SE	OR (95% CI)	*p* Value
MAFLD (yes vs. no)	2.1	0.30	3.5 (3.1–6.5)	<0.001
BMI	1.9	0.05	2.5 (1.5–3.4)	0.045
IL-6	2.0	0.60	2.2 (1.8–2.7)	0.03

## Data Availability

Data regarding investigated patients and statistical analysis will not be made publicly available.
